# Inhibition of the IFN Response by Bluetongue Virus: The Story So Far

**DOI:** 10.3389/fmicb.2021.692069

**Published:** 2021-06-08

**Authors:** José Manuel Rojas, Miguel Avia, Verónica Martín, Noemí Sevilla

**Affiliations:** Centro de Investigación en Sanidad Animal (CISA-INIA), Centro Nacional Instituto de Investigación y Tecnología Agraria y Alimentaria, Consejo Superior de Investigaciones Científicas (CSIC), Madrid, Spain

**Keywords:** orbivirus, BTV-NS3, BTV-NS4, double-stranded RNA, STAT1, STAT2, TBK1, IFNAR^–/–^ mice

## Abstract

Bluetongue virus (BTV) is the prototypical orbivirus that belongs to the *Reoviridae* family. BTV infection produces a disease in ruminants, particularly in sheep, that results in economic losses through reduced productivity. BTV is transmitted by the bite of *Culicoides* spp. midges and is nowadays distributed globally throughout subtropical and even temperate regions. As most viruses, BTV is susceptible to the IFN response, the first line of defense employed by the immune system to combat viral infections. In turn, BTV has evolved strategies to counter the IFN response and promote its replication. The present review we will revise the works describing how BTV interferes with the IFN response.

## Introduction

The interferon (IFN) system is present in all vertebrates and is central to antiviral immunity. Cells can respond to viral infection by secreting IFNs that warn the neighboring cells of the ongoing risk and trigger a programming that renders cells refractory to infection. Cell exposure to IFN prompts the expression of over 2,000 interferon-stimulated genes (ISG) and represses the expression of nearly 1,500 genes in humans that ultimately promotes an antiviral state (Shaw et al., 2017). The antiviral state is essential for protection against viral infection as defects in the signaling pathways involved either in IFN induction or signaling lead to increased susceptibility to viral pathogens. IFN responses are also critical for the development of an adequate adaptive immunity.

Cells can sense viral presence through pattern recognition receptors (PRRs) that recognize pathogen-associated molecular patterns (PAMPs) that include pathogen-related nucleic acids, proteins and carbohydrates (Goubau et al., 2013; Ma and Damania, 2016; McFadden et al., 2017; Abe et al., 2019). Typically, one of the main PAMPs recognized during viral infection consists in the viral nucleic acids and their intermediates produced during viral replication (Chan and Gack, 2016; Rojas et al., 2021a). Activation of PRRs by PAMPs triggers several signaling cascades among which are included pathways that result in IFN induction. Once IFNs are produced, they can signal to neighboring cells and trigger the antiviral state. IFNs can be classified in three groups according to the receptors they utilize for signaling (reviewed in Pestka et al., 2004). Type I IFNs (IFN-I), which includes IFN-αs and IFN-β, signal through the ubiquitously expressed type I IFN receptor (IFNAR). Type II IFN (IFN-II), which only includes IFN-γ and is mostly produced by immune cells, signals through the type II IFN receptor (IFNGR). Finally type III IFNs (IFN-III), or IFN-λs, signal through the type III IFN receptor that is mostly expressed on epithelial cells. IFN-I and IFN-III range of activities is mostly centered on antiviral immunity, whereas IFN-γ is mostly involved in the modulation of adaptive immunity, although it also possesses antiviral properties (McNab et al., 2015).

The aim of the present review is not to fully describe these activating pathways but to concisely address these in the context of bluetongue virus (BTV) infection. Extensive reviews on virus sensing and the events leading to IFN induction and signaling have been published elsewhere should the reader seek more in depth information (Goubau et al., 2013; McNab et al., 2015; Chan and Gack, 2016; Ma and Damania, 2016; McFadden et al., 2017; Abe et al., 2019; Rojas et al., 2021a).

## The IFN System: From IFN Induction to ISG Production

**FIGURE 1:**
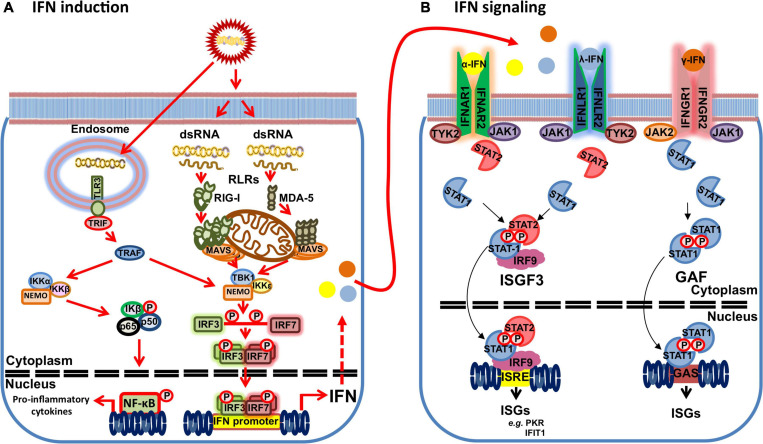
Schematic of the IFN response to dsRNA viruses. **(A)** dsRNA motifs can be recognized by retinoic acid-inducible gene-I (RIG-I) like receptors (RLRs) (RIG-I and MDA-5) in the cytoplasm or Toll-like receptor (TLR) 3 in the endosomes that trigger IFN and proinflammatory cytokine production. Activated RLR are recruited to mitochondrial antiviral signaling (MAVS) protein, which in turn recruit tumor necrosis factor receptor-associated factors (TRAF). TLR3 signals through its adaptor protein Toll/IL-1R domain-containing adaptor inducing IFN-β (TRIF) to activate TRAFs. TRAFs promote the activation of the TANK binding kinase 1 (TBK1) complex [composed of TBK1, IκB kinase ε (IKKε) and NF-κB essential modulator (NEMO)] leading to TBK1 activation that mediates phosphorylation and homodimerization of the IFN regulatory factors (IRF) 3/7. Activated IRF3/7 dimers translocate to the nucleus to trigger IFN gene transcription. TRAF activation also promote the activation of the IKK complex (composed of IKKα and β, and NEMO) that activates NF-κB and the transcription of pro-inflammatory cytokines. **(B)** IFNs signals through their heterodimeric surface receptors (IFNAR1 and 2 for α-IFN; IFNLR 1 and 2 for λ-IFN; and IFNGR 1 and 2 for γ-IFN) that lead to activation of intracellular Janus Kinase (JAK)/signal transducer and activator of transcription (STAT) pathways and to the transcription of IFN-stimulated genes (ISGs). Binding of α-IFN to its receptor activates the JAK kinases JAK1 and TYK2, which in turn leads to the phosphorylation of STAT1 and STAT2. Phosphorylated STAT1 and STAT2 dimerizes and binds to IRF9 to form the transcriptional factor complex IFN stimulated gene factor (ISGF) 3 that translocates to the nucleus to bind the IFN-response elements (ISRE) in ISG promoters and drive the expression of ISG products, such as protein kinase R (PKR) or interferon-induced proteins with tetratricopeptide repeats (IFIT) 1. λ-IFN signaling follows this same canonical pathway as α-IFN signaling. γ-IFN signals through its receptor by activating the JAK kinases JAK1 and JAK2 that leads to STAT1 phosphorylation and homodimerization into the transcription factor termed the gamma interferon-activated factor (GAF). GAF then binds gamma interferon-activated site (GAS) to drive the transcription of ISGs dependent on γ-IFN.

As previously mentioned, cells can sense viral genome through PRRs. These can be broadly classified in five groups: Toll-like receptors (reviewed in Finberg et al., 2007), retinoic acid-inducible gene-I (RIG-I) like receptors (RLRs) (reviewed in Rehwinkel and Gack, 2020), nucleotide oligomerization domain-like receptors (NLRs) (reviewed in Jacobs and Damania, 2012); non-RLR DEXD/H-box helicases (reviewed in Chow et al., 2018); and cytosolic DNA sensors (reviewed in Ma et al., 2018). Double-stranded RNA (dsRNA) viruses, such as BTV, are typically sensed by RLRs in the cytoplasm (Figure 1). These include RIG-I and melanoma differentiation associated factor 5 (MDA-5), both of which sense different dsRNA motifs (Rehwinkel and Gack, 2020). While RLRs are ubiquitously expressed, TLR expression is mostly restricted to cells of the hematopoietic linage. TLR3 is the prototypical dsRNA sensor that recognizes this genetic material in endosomes (Finberg et al., 2007). Recognition of viral genetic material triggers a signaling cascade that typically leads to IFN-I induction and production of pro-inflammatory cytokines. RIG-I and MDA-5 possess two caspase activation and recruitment domains (CARD) that are liberated upon RLR activation. The released RLR CARDs can thus interact with the CARD of the mitochondrial antiviral signaling (MAVS) protein. This forms prion-like aggregates that are essential for MAVS recruitment of the tumor necrosis factor receptor-associated factors (TRAF) responsible for downstream signaling (Ren et al., 2020). TRAFs then promote the activation of the TANK binding kinase 1 (TBK1) complex [composed of TBK1, IκB kinase ε (IKKε), and NF-κB essential modulator (NEMO)], which induce IFN induction, and the IKK complex (composed of IKKα and β, and NEMO) that activates NF-κB and the transcription of pro-inflammatory cytokines (Yu et al., 2012). The activated TBK1 complex can then mediated the phosphorylation and homodimerization of the IFN regulatory factors (IRF) 3/7 that consequently translocate to the nucleus to trigger the transcription of early IFN response genes, such as IFN-I.

The production of IFN-I acts as a warning signal on neighboring cells that prompts an antiviral state that protects from infection. IFN-I signal through the heterodimeric IFNα receptor (IFNAR) composed of α and β transmembrane subunits (Pestka et al., 2004). IFN binding to the receptor elicits a signaling cascade through Janus kinase family (JAK)/signal transducer and activator of transcription (STAT) pathways (Figure 1; Majoros et al., 2017; Nan et al., 2017). IFN-I binding leads to the sequential phosphorylation of JAK kinases Jak1 and Tyk2, which in turn phosphorylate STAT1 and STAT2. Phosphorylated STAT1 and STAT2 heterodimerize and bind to IRF9 to form the transcriptional factor complex IFN stimulated gene factor (ISGF) 3. ISGF3 then translocates to the nucleus and binds the IFN-response elements (ISRE) in ISG promoters leading to the expression of ISG products. Similarly, IFN-II signaling is transduced through JAK/STAT pathways. IFN-II binding to its receptor triggers Jak1 and Jak2 phosphorylation, which in turn leads to STAT1 phosphorylation. Phosphorylated STAT1 homodimerizes and translocates to the nucleus to bind gamma interferon-activated site (GAS) and thus drive the transcription of ISGs dependent on IFN-II (Majoros et al., 2017).

ISG expression provides the cell with mechanisms to combat the viral infection as a result of IFN stimulation (reviewed in Schneider et al., 2014). IFN responses also repress some gene expression (Shaw et al., 2017), although these are not as widely studied and will not be discussed in the present review. It is also noteworthy that ISG expression differs between species (Shaw et al., 2017), and this should be considered when analyzing the IFN response using *in vitro* tools based on species that differ from the natural host of the disease. ISG products promote multiple aspects of the cell antiviral response. They can among other things cooperate in PRR recognition of viral PAMPs, block virus entry, stabilize signaling complexes, hinder viral capsid formation, or impair virion exit from infected cells. Some ISG products also modulate the IFN response to prevent the toxicity of these potent immune mediators. Among ISGs, the protein kinase R (PKR) that detects cytosolic dsRNA belongs to one of the so-called classical ISG pathways. The activated form of PKR regulates translation initiation by mean of phosphorylation of the alpha subunit of the eukaryotic initiator factor eIF2 (Williams, 1999). PKR also participates in other mechanisms in the antiviral state, such as apoptosis induction (Gil and Esteban, 2000), regulation of IFN-β synthesis and of NF-κB-mediated pro-inflammatory cytokine pathway (Kirchhoff et al., 1995; Kumar et al., 1997; Chu et al., 1999), or modulation of STAT1 activity in the IFN-I signaling pathway (Wong et al., 1997). Overall, PKR recognition of dsRNA limits viral replication through these mechanisms. Another family of ISGs with anti-viral functions are the interferon-induced proteins with tetratricopeptide repeats (IFITs) which includes four members in humans (IFIT1, 2, 3, and 5) and three in mice (IFIT1, 2, and 3) (reviewed in Vladimer et al., 2014). The transcription of these genes is rapidly increased after activation by IFN signaling but also after viral PAMPs recognition. IFIT1 detects the absence of 2′-O-methylation on RNAs species, a methylation present in eukaryotic mRNA but lacking in some viral RNA (Habjan et al., 2013). IFIT1 also recognizes the 5′-triP end present in some viral RNA (Abbas et al., 2013). IFIT1 can sequester viral RNAs to prevent their transcription and also inhibit the translation initiation of these IFIT1-bound RNA species by the translation initiation factor 3, thus providing the cells with means to limit viral replication.

Thus the IFN response is mediated by a complex system of PAMP recognition triggering signaling pathways that lead to the translation of effector ISG products that limit viral replication. Viruses can often interfere at multiple points in these pathways so that they can replicate and continue their infectious cycle in spite of the IFN response. In this review, we will revise the current knowledge on the interactions of Bluetongue virus (BTV) with the mammalian host IFN response.

## Bluetongue Virus (BTV)

Bluetongue virus (BTV) is the prototypical orbivirus that belongs to the *Reoviridae* family. Orbiviruses are arthropod-borne viruses (arboviruses) which replicates in arthropod and vertebrate hosts (reviewed in Attoui et al., 2016). Infection of the vertebrate host is usually mediated by the bite of ticks or hematophagous insects. In the case of BTV, the virus is transmitted to the ruminant host by the bite of infected *Culicoides* spp. midges (Baylis et al., 2008). It produces an economically important disease of compulsory declaration to the OIE (World Organization for Animal Health) that limits productivity in small ruminants (reviewed in Rushton and Lyons, 2015). During outbreaks, high mortality rates can occur in naïve herds, whereas in endemic regions limitation on animal movement, production losses (e.g., wool breaks), and reduced fertility can hamper profitability. The existence of multiple serotypes that confer little cross-protection in terms of sterilizing immunity between serotypes, and the possibility for reassortant formation when multiple BTV strains are co-circulating within one territory complicates the control of this disease. As such, BTV has established itself as an endemic disease in the southern European latitudes with frequent incursions in northern latitudes in the late summer and autumn (Baylis et al., 2017).

**FIGURE 2:**
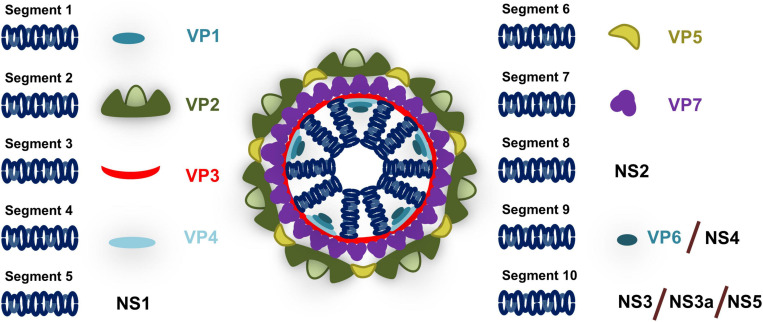
Schematic of BTV particle. BTV genome consists of 10 segments that encodes for structural proteins (VP) present in the viral particle and non-structural proteins (NS) that are expressed during infection. Segment 1 encodes for the RNA polymerase VP1; Segment 2 for the outer capsid protein VP2; Segment 3 for the inner core protein VP3; Segment 4 for the RNA capping enzyme and methyl transferase VP4; Segment 5 for NS1 which is responsible for the formation of tubular structures during infection; Segment 6 for the outer capsid protein VP5; Segment 7 for the inner core protein VP7; Segment 8 for the RNA binding protein NS2 that is involved in inner core formation; Segment 9 for RNA helicase VP6 and for NS4 which acts as an IFN antagonist; and Segment 10 for NS3 (and its isoform NS3a) which is involved in virion egress and IFN antagonism and putatively for NS5 that could be involved in cell shutoff.

BTV possesses a segmented genome consisting of 10 dsRNA segments encoding for 7 structural and 4–5 non-structural proteins (Figure 2). The BTV particle consists of an outer capsid formed by VP2 and VP5 proteins that mediates cell attachment and entry, and an inner capsid (core) formed by VP7 and VP3 proteins that encapsulated the genetic material as well as the RNA polymerase VP1, the RNA capping enzyme and methyl transferase VP4, and the RNA helicase VP6 (reviewed in Roy, 2005, 2017). The virus also encodes for at least four non-structural proteins termed NS1 to NS4. NS1 forms tubular structures in the cytoplasm and promotes viral protein expression through a mechanism that involves transition from the NS1 tubular form to an active non-tubular form (Boyce et al., 2012; Kerviel et al., 2019). NS2 is an RNA binding protein which is the major component of viral inclusion bodies (VIB) and plays a critical role in the inner core formation (Kar et al., 2007). NS3 (and the shorter isoform NS3a which is encoded by an in-frame alternate ORF) is involved in virion egress (Han and Harty, 2004; Wirblich et al., 2006; Celma and Roy, 2009; Labadie et al., 2019). NS3 has also been identified as an antagonist of the IFN response (Chauveau et al., 2013; Avia et al., 2019) as will be discussed in this review. NS4 is encoded by an alternative ORF in segment 9 (Belhouchet et al., 2011; Ratinier et al., 2011) and also acts as an IFN antagonist (Ratinier et al., 2016). Finally an alternate ORF has been identified in segment 10 which could encode for an additional non-structural protein NS5 (Stewart et al., 2015), although its expression during infection and role has yet to be clarified.

## BTV and the IFN Response

### BTV Is Sensitive to the IFN Response but Can Overcome It in Ruminants

Like for most viruses, BTV replication can be impaired *in vitro* by exogenous addition of IFN-I. Doceul et al. (2014) showed that addition of IFN-β to the human alveolar epithelial A549 cells and to Hela cells impaired viral replication in 2 BTV serotypes. Treatment of ovine CPT-Tert cells with universal type I IFN or the ovine type I IFN-τ prior to BTV-1 or BTV-8 infections also impaired viral replication (Ratinier et al., 2011). BTV is, however, still capable of replication in presence of IFN (particularly in cells derived from its natural host) (Ratinier et al., 2011), indicating that the virus possesses mechanisms to overcome IFN effects. There is evidence of the protective role of IFN-I during BTV infections *in vivo*. In murine models, hematopoietic progenitors and dendritic cells (DC) are highly susceptible to BTV infection in absence of IFN-I receptor, whereas in the presence of the receptor they resist infection (Rodriguez-Calvo et al., 2014). The protective role of IFN-I during BTV infections is also clearly exemplified by the susceptibility to infection in mice with impaired IFN-I signaling either through knock-out of type I IFN receptor β chain gene [IFNAR^(–/–)^ mice] (Calvo-Pinilla et al., 2009a) or through antibody blockade of the receptor (Saminathan et al., 2020). Indeed, the transgenic IFNAR^(–/–)^ murine model is now widely used to assess the protective capacity of candidate vaccines for BTV (Calvo-Pinilla et al., 2009b, 2012; Martin et al., 2015; Marín-López et al., 2018; Rojas et al., 2021b), as it recapitulates some of the effects of acute BTV infection in the natural host (Marín-López et al., 2016). There is also evidence that IFN-I limits BTV replication in ruminants. Early induction of IFN-I probably limits BTV replication in infected calves, as BTV titer increases once the IFN levels subside in serum (MacLachlan and Thompson, 1985). Diphasic BTV viremia peaks have also been reported in sheep infection, and this coincided with the IFN-I response. Initial viremia appears suppressed by IFN-I induction, but then peaks again once the IFN-I response diminishes (Foster et al., 1991). These data clearly hint at a protective role for IFN-I against BTV in the natural host. BTV is nonetheless capable of replicating in the natural host in spite of a concomitant IFN response. Melzi et al. (2016) showed that BTV presence in the lymph node caused an early induction of the ISG MX1, yet the viral infection progressed in sheep. Indeed, susceptibility to BTV infection could partially be explained by differences in ISG induction between species (Shaw et al., 2017). This is exemplified by the capacity of BTV to infect IFNAR^(–/–)^ mice but not wild-type counterparts indicating that mice naturally express a range of ISGs that counter BTV replication, while ruminant ISGs are unable to fully counter this.

### BTV Infection Induces IFN

IFN induction during BTV infection was first described in cell culture in 1969 using an attenuated BTV-10 strain and infecting murine embryonic cells (Huismans, 1969). Several groups later confirmed IFN induction in ruminant cells (Rinaldo et al., 1975; Coen et al., 1991; Russell et al., 1996; Chauveau et al., 2012) and in other mammalian cells (Jameson and Grossberg, 1979, 1981; Fulton and Pearson, 1982). *In vivo*, BTV has been described as a good inducer of IFN in mice (Jameson et al., 1978). BTV infection in cattle and sheep leads to IFN production (MacLachlan and Thompson, 1985; Foster et al., 1991; Ruscanu et al., 2012). Comparative analysis of the IFN-I response in bovine and ovine endothelial cells does not hint at major differences in IFN induction *in vitro* (Russell et al., 1996). Indeed, ovine cells appear to produce higher amount of IFN-I than their bovine counterpart. In spite of this IFN response, BTV was still capable of replicating productively in endothelial cells (Russell et al., 1996), showing that the virus possesses mechanisms to overcome the IFN response.

It should be noted that BTV infection not only triggers IFN-I response in ruminants, it also elicits the production of proinflammatory cytokines *in vivo* (Channappanavar et al., 2012; Umeshappa et al., 2012; Sanchez-Cordon et al., 2015; Rojas et al., 2017). *In vitro* infection of bovine cells targeted by BTV, such as endothelial cells or macrophages showed an increase in characteristic pro-inflammatory cytokines, such as TNF-α, IL-1β, and IL-8 (Drew et al., 2010). The pro-inflammatory cytokine and IFN response generated by BTV infection that on the one hand probably limits BTV replication could, on another hand, also account for some of the *in vivo* inflammatory vascular lesions that are typically produced by the disease. This is often referred to as a “cytokine storm” and accounts for some of the pathogenic effects on vasculature that BTV triggers (Howerth, 2015). Pro-inflammatory mediators triggered by the infection can modulate BTV infection of endothelial cells and this is also dependent on the origin of the endothelial material (DeMaula et al., 2001, 2002a, 2002b). These studies also showed differential sensitivity to cell death of ovine lung microvascular endothelial cells upon exposure to proinflammatory mediators and BTV when compared to bovine counterparts, which could explain some of the BTV pathogenic effects on sheep vasculature.

Early BTV infection events have been associated with DC infection that allows spreading of the virus to the lymphoid tissue. Hemati et al. (2009) elegantly showed using cannulation of the afferent lymph duct in sheep that BTV infection of conventional DC (cDC) in the skin contribute to the dissemination of the virus to the lymph node. cDC infection did not appear to affect DC function *in vitro* as DC activation and adequate antigen presentation to T cells still occurred in infected cDCs. However, *in vivo* experiments have indicated that BTV infection has the capacity to disrupt follicular DC function in the lymph node (Melzi et al., 2016). Melzi et al. (2016) confirmed that BTV disseminates to the lymph node via the lymph, and showed that this triggered as IFN response as demonstrated by the induction of the ISG MX1 in the lymph node of infected sheep. In spite of this IFN response, BTV still disrupted the humoral response, delaying the antibody response and decreasing the antibody avidity to a model antigen, further demonstrating that BTV can replicate in presence of IFN-induced antiviral mechanisms (Melzi et al., 2016). Although cDC are probably the main cell type responsible for dissemination, they are unlikely to be the main source of *in vivo* IFN. Ruscanu et al. (2012) showed that purified plasmacytoid DC (pDC) from sheep responded to BTV by producing IFN-I, while cDC were poor producers of IFN-I. UV-inactivated BTV also induced IFN-I indicating that the viral particle possesses PAMPs that can be recognized by pDCs even in the absence of replication. Subsequent microarray analysis of circulating pDCs in infected sheep confirmed that these cells acquire a pro-inflammatory profile upon infection that includes IFN-I activation (Ruscanu et al., 2013); thus pointing at this cell type as a plausible suspect for triggering the immune-related pathogenesis in BTV infections. Interestingly, pDCs lose their pro-inflammatory profile within the lymph nodes while maintaining their IFN responsiveness, which illustrate the complex mechanisms that the microenvironment plays in modulating DC responses. Taken together these studies demonstrate that BTV is readily detected upon infection and that IFN-I responses are mounted.

This brings us to the next question: which BTV PAMPs are recognized by the innate immune system? As previously discussed, BTV replication is not necessary to trigger the IFN response of pDCs, but it increased the response of these cells (Ruscanu et al., 2012). This indicates that the BTV particle possesses PAMPs readily recognizable by PRRs. It is well-documented that the dsRNA of *Reoviridae* is sufficient to trigger IFN production (Tytell et al., 1967; Abad and Danthi, 2020). BTV dsRNA can indeed induce IFN responses in mice (Huismans, 1969), and transfection with BTV dsRNA can trigger the activity of the IFN-β promoter (Vitour et al., 2014). BTV dsRNA represents therefore a motif recognizable by cellular PRRs. Given that BTV replication has been associated with increased IFN-I production and activation of the IFN-β promoter (Chauveau et al., 2012; Ruscanu et al., 2012), it is probable that viral replication intermediates are also detected by PRRs and this enhances the IFN response. Whether some BTV proteins could also be detected by PRRs, similarly to the hemagglutinin of some morbilliviruses that activates TLR2 (Bieback et al., 2002; Rojas et al., 2021c), is not known.

**FIGURE 3:**
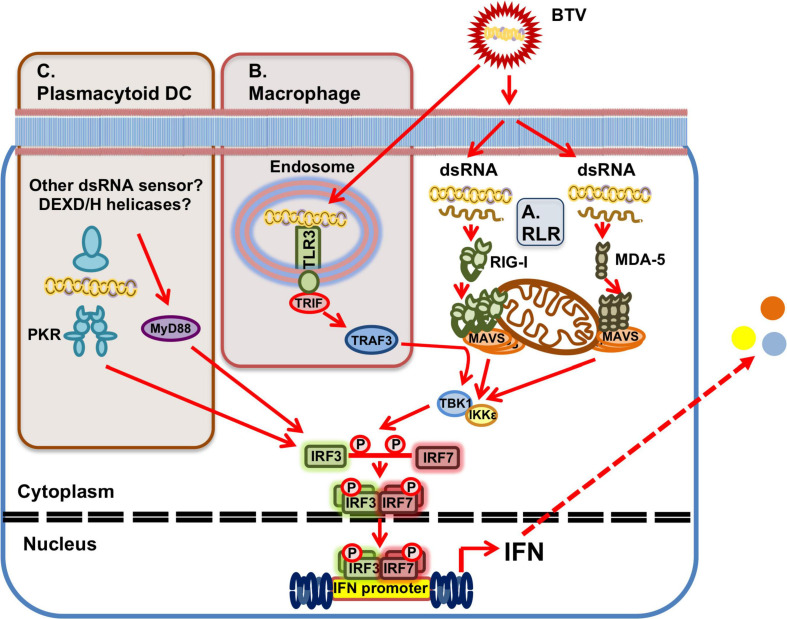
BTV sensing by PRRs. **(A)** Ubiquitously expressed RLRs can detect BTV dsRNA presence in the cytoplasm. RIG-I and MDA-5 are involved in BTV recognition and this triggers a signaling cascade that results in IFN induction through activation of the MAVS/TBK1 pathway. **(B)** In macrophages, TLR3 in endosomes can also recognize BTV dsRNA resulting in IFN induction and the generation of an antiviral state. **(C)** In plasmacytoid DC, which do not express TLR3, PKR is involved in dsRNA recognition in the cytoplasm and IFN induction. IFN induction in these cells is also dependent on the adaptor MyD88 for IFN induction, but recognition is independent of TLR7. Whether other dsRNA sensors, such as DEXD/H-box helicases are involved upstream of MyD88 for dsRNA sensing in pDCs has yet to be determined.

At a molecular level, BTV has been shown to activate the transcription factors NF-κB, IRF3, and IRF7 early in the infection in HeLa cells (Stewart and Roy, 2010) confirming that BTV infections have the potential to trigger pro-inflammatory responses mediated by NF-κB and IFN responses mediated by IRF3/7. Indeed, Ratinier et al. (2016) showed that BTV infection of A549 cells triggered nuclear translocation of the IRF3 and NF-κB complexes. The cytosolic RNA sensors RIG-I and MDA-5 are involved in the recognition of BTV during infection (Chauveau et al., 2012; Figure 3). Using siRNA targeting RIG-I and MDA-5, Chauveau et al. (2012) showed in A549 cells that IFN-β induction depends on these RLRs. Moreover, upregulation of RIG-I and MDA-5 also impaired BTV replication, demonstrating that these PRRs participate in the establishment of the antiviral state in BTV infections. Chauveau et al. (2012) confirmed the activation of NF-κB and IRF3 upon BTV infection, and showed this activation was dependent on MAVS signaling upstream of the transcription factors and downstream of the cytosolic RNA sensors RIG-I and MDA-5.

BTV PAMP sensing will also probably vary depending on cell types. While RLR are ubiquitously expressed, TLRs are mostly expressed in immune cells. In human primary macrophage cultures, BTV activated IFN-I responses via TLR3 recognition (Dai et al., 2015; Figure 3). Although BTV is unable to productively infect these cells, human macrophage treatment with live BTV or UV-inactivated BTV triggered an antiviral state, indicating that the dsRNA sensor TLR3 can detect BTV genetic material in macrophages (Dai et al., 2015). As previously discussed, ovine pDCs produce IFN-I when infected with BTV or when they internalized UV-inactivated virus (Ruscanu et al., 2012). Ruscanu et al. (2012) found that IFN-I induction required internalization through endosomal compartment for both the live and inactivated virus, which hinted at TLR-mediated activation of IFN-I. However pDC do not express TLR3 (Liu, 2005), the typical sensor for dsRNA in endosomes, and thus other RNA sensors are probably implicated in BTV PAMPs recognition in these cells. Ruscanu et al. (2012) showed in ovine pDCs that IFN-I induction signaling occurred at least partly through the adaptor myeloid differentiation factor 88 (MyD88), which is usually associated to TLR signaling, but was independent of the RNA sensor TLR7 (Figure 3). Pharmacological inhibition of PKR reduced the IFN-I response to BTV, suggesting that this sensor is involved in BTV recognition in these cells (Ruscanu et al., 2012). Other RNA sensors could also be implicated in BTV sensing, such as members of the DEXD/H-box helicase family. The cytosolic RNA sensor DHX33 has been shown to recognize reoviral dsRNA and activate the inflammasome in primary human macrophages (Mitoma et al., 2013), while other members of this family have been shown to detect dsRNA in myeloid DCs (Zhang et al., 2011). The exact mechanisms of BTV recognition in pDC are yet to be fully elucidated and may implicate several RNA recognition pathways. Since pDC have been identified as the main IFN-I producer cells in early infection (Ruscanu et al., 2012), identification of the exact mechanism of IFN-I induction in these cells could provide important clues on the host-pathogen interactions.

## BTV Employs Multiple Proteins to Impair the Host IFN Response

As previously mentioned IFNAR^(–/–)^ mice are susceptible to BTV infection (Calvo-Pinilla et al., 2009a) and thus can be used to evaluate the pathogenicity of BTV strains. This indicates that BTV virulence is at least partly dependent on the host IFN response. Studies in virulence factors influencing BTV pathogenesis have implicated several proteins encoded by the viral genome (Caporale et al., 2011; Celma et al., 2014; Janowicz et al., 2015). The outer capsid proteins VP2 and VP5, which are involved in viral particle entry in mammalian cells, and the RNA polymerase VP1 have been identified as determinants of virulence in BTV-8 when a multiple passaged strain was compared to the parental strain (Janowicz et al., 2015). These changes probably reflect the adaptation of the virus strain to tissue culture conditions, which in turn led to reduced infectivity in the IFNAR^(–/–)^ mouse model. In these studies, NS3 appears to be a major player in virulence as the sole introduction of the NS3-encoding segment from the multiple-passaged strain into the pathogenic parental strain significantly impaired pathogenesis. Indeed, using reverse genetics, NS3 was also shown to modulate BTV replication kinetics and cytopathic effects in mammalian cells (Ftaich et al., 2015). The presence of a proline residue at position 24 reduced NS3 protein half-life, which in turn decreased the virulence in IFNAR^(–/–)^ mice of a reassortant virus carrying this segment 10 (Ftaich et al., 2015). NS3 is, however, not solely responsible for pathogenesis, as a reassortant virus that expresses VP1, VP2, VP5 and NS3 from the pathogenic strain on the backbone of the non-pathogenic multiple passage strain did not revert to the pathogenic phenotype (Janowicz et al., 2015). Indeed, additional expression of capping enzyme and methyl transferase VP4, inner capsid protein VP7, and RNA helicase VP6 and non-structural protein NS4 encoded by segment 9 was essential to reverse the attenuation of the multiple-passaged strain (Janowicz et al., 2015). It thus appears that BTV uses multiple proteins to impair host responses and promote its replication.

**FIGURE 4:**
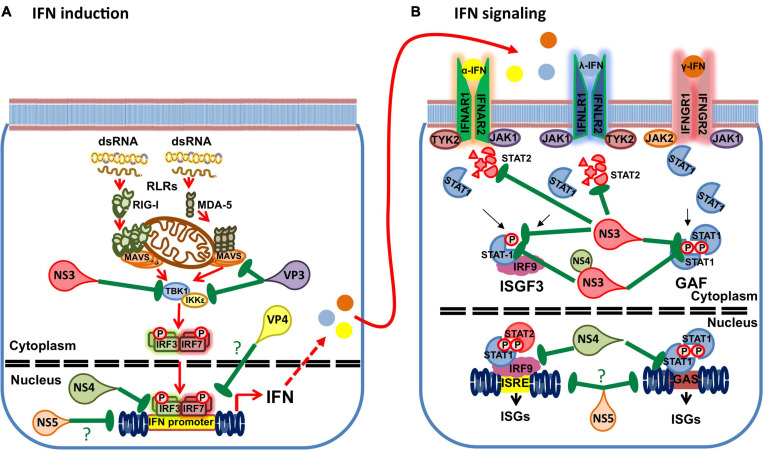
Interference of BTV gene products with the IFN response. **(A)** BTV uses multiple gene products to impair IFN induction. NS3 has been shown to impair TBK1 activation by targeting the ubiquitin-binding protein Optineurin responsible for TBK1 translocation to the Golgi apparatus, where TBK1 activation by trans-autophosphorylation takes places. NS4 has been shown to impair IFN promoter gene activity in reporter assays. VP3 has been shown to interact with MAVS and IKKε to interfere with IFN induction. VP4 has been reported to interfere with IFN induction, but the mechanism has yet to be clarified. Finally the putative NS5 has been shown to interfere with the activity of multiple promoters including the IFN-β promoter. **(B)** BTV also uses multiple gene products to interfere with IFN signaling. NS3 can target STAT2 for degradation through an autophagic/lysosomal pathway. NS3 can also interfere with STAT1 phosphorylation. NS4 and NS3 appear to coordinately impair STAT1 phosphorylation by binding to STAT1 SH2 domain. NS4 can also interfere with the ISRE and GAS promoter activity. Finally, the putative NS5 can impair the activity of multiple promoters and thus possibly impair ISRE and GAS promoter activity. (?) denotes unknown or speculative mechanisms.

Using reporter assays, BTV has been shown to impair IFN induction (Chauveau et al., 2013; Avia et al., 2019). BTV infections have also been shown to inhibit IFN-I and -II signaling by preventing STAT1 phosphorylation and subsequent translocation to the nucleus (Doceul et al., 2014; Avia et al., 2019). Doceul et al. (2014) found that BTV infection reduced JAK1 and TYK2 expression while Avia et al. (2019) found that STAT2 expression decreased in infected cells. At least three BTV gene products are involved in impairing IFN responses (Figure 4). In the next section we will discuss the current knowledge of the mechanisms employed by BTV to impair the host IFN response.

### BTV-NS3

BTV-NS3 is the main viral protein involved in virion egress in insect and mammalian cells (Hyatt et al., 1993; Beaton et al., 2002; Labadie et al., 2020). NS3 is a transmembrane protein that is likely synthesized in the ER and traffics through the Golgi apparatus to reach the plasma membrane (Wu et al., 1992; Bansal et al., 1998; Bhattacharya et al., 2015; Labadie et al., 2020). NS3 C-terminal region interacts with the outer BTV capsid protein VP2 which allows virion transport to the cell surface (Celma and Roy, 2009). NS3 presence in virus inclusion bodies has recently been demonstrated (Mohl et al., 2020), and its correct trafficking to the cell membrane is essential for virus maturation and release (Labadie et al., 2019). NS3 has also been identified as a viroporin as it can assemble in the form of pores in membranes (Chacko et al., 2015), thus probably facilitating the release of the BTV particles from the cell. Indeed, given the role of NS3 in virus exit, recombinant BTV particles deficient in the NS3-encoding segment have been proposed as a disabled infectious single animal vaccine (van Rijn et al., 2017). Recently, NS3 was also shown to activate the MAPK/ERK pathway probably to promote cell survival and increase protein translation, thus increasing virus replication (Kundlacz et al., 2019).

Besides its role in virion release, NS3 has been identified as a virulence factor (Janowicz et al., 2015), and could be involved in cellular host protein shutdown. Stewart et al. (2015) showed that NS3 transfection could decrease basal luciferase activity driven by a variety of promoters. Avia et al. (2019) also found that transfection with high amounts of NS3-encoding plasmid can sometimes produce a similar effect on luciferase basal activity. Finally, NS3 has also been described as an IFN antagonist in several studies (Chauveau et al., 2013; Doceul et al., 2014; Avia et al., 2019; Li et al., 2021). In 2013, Chauveau et al. (2013) showed using reporter assays that NS3 interferes with RLR signaling, thus blocking the transcription of IFN and NF-κB-stimulated genes. This inhibition occurred downstream of RLR recognition and upstream of IKKε activation. In a subsequent study, NS3 was found to interfere with TBK1 activation by interacting with optineurin in the Golgi apparatus (Pourcelot et al., 2016). Upon RLR or TLR3 activation, TBK1 is ubiquitinated and transported to the Golgi apparatus where TBK1 complexes are activated by trans-autophosphorylation, which eventually leads to IRF3 phosphorylation and IFN induction (Pourcelot et al., 2016). Optineurin is an ubiquitin binding protein that recruits TBK1 to the Golgi apparatus and facilitates the formation of the TBK1 activation complexes (Ryan and Tumbarello, 2018). By binding optineurin in the Golgi apparatus, BTV-NS3 is therefore capable of dampening TBK1 phosphorylation and consequently IFN induction (Pourcelot et al., 2016). Interestingly, BTV-NS3 is also ubiquitinated (Avia et al., 2019) and it could be speculated that NS3 could act as a competing substrate for optineurin binding in the Golgi apparatus. Further work in this area will be necessary to determine the exact mechanism of NS3 interference with optineurin activity.

NS3 not only interferes with IFN-I induction, it also inhibits IFN-I and IFN-II signaling. NS3 transfection in cell lines was shown to impair STAT1 phosphorylation and translocation to the nucleus (Avia et al., 2019; Li et al., 2021). Importantly, infection with a reverse genetic BTV lacking the segment encoding for NS3 could only partially impair STAT1 phosphorylation and translocation indicating that NS3 is not solely responsible for this interference mechanisms (Avia et al., 2019). Indeed a collaborative role of another non-structural protein (NS4) with NS3 has been proposed as a mechanism that enhances STAT1 interference (Li et al., 2021). While Avia et al. (2019) found that NS3 did not interact with STAT1 in immunoprecipitation studies; Li et al. (2021) reported that NS3 interact with STAT1 SH2 domain. These discrepancies could be due to differences in the immunoprecipitation procedures: Avia et al. (2019) assessed endogenous STAT1 protein levels precipitated with NS3, whereas Li et al. (2021) used assessed this interaction using cells transfected with both NS3 and STAT1. It should be noted that these studies also employed gene products cloned from different BTV serotypes (BTV-8 for Avia et al., 2019 and BTV-1 for Li et al., 2021), which could also contribute for the observed differences. Overall, NS3 interferes with STAT1 phosphorylation and nuclear translocation thereby impairing IFN signaling, although the mechanism has yet to be fully elucidated.

**FIGURE 5:**
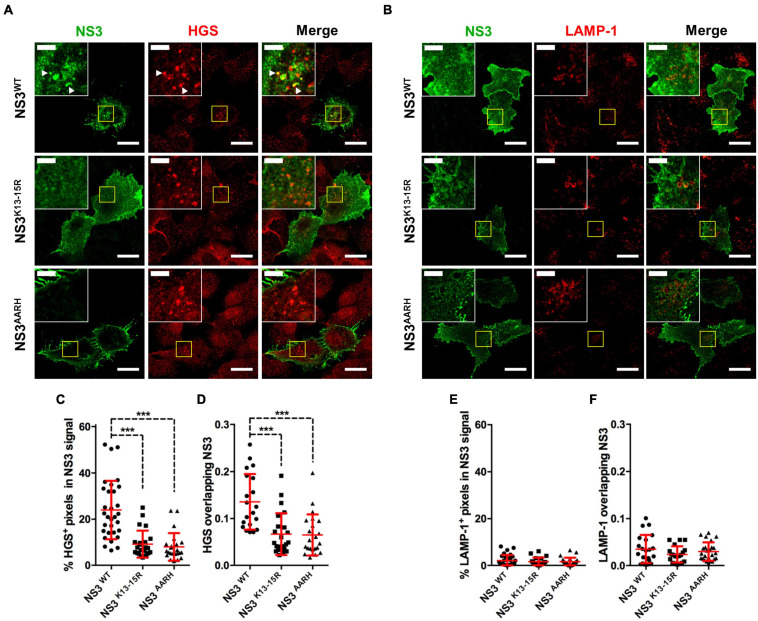
NS3 traffics through the MVB. **(A,B)** Representative immunofluorescence staining of Vero cells transfected with plasmids expressing Flag-tagged NS3^*WT*^, NS3^*K*13–15R^ (ubiquitination site mutation), or NS3^*AARH*^ (E3 ligase binding domain mutation) and stained for NS3 with anti-FLAG tag antibodies [Cell Signaling, #14793 (D6W5B)], and for **(A)** the MVB marker HGS (Abcam, ab72053) or **(B)** the lysosome marker LAMP-1 (BioLegend, 328612). Scale bar = 20 μm. Inset shows selected areas and arrowheads indicate pixel colocalization. Inset scale bar = 5 μm. **(C–F)** Pixel colocalization assessment using ImageJ software (http://rsbweb.nih.gov/ij/ US National Institutes of Health). Mean ± SD percentage area per cell of **(C)** HGS + or **(E)** LAMP-1 + pixels in NS3 signal. Manders’ coefficient per cell for **(D)** HGS or **(F)** LAMP-1 signal overlapping NS3 determined by the ImageJ JACOP plugin are shown. Cumulative data from 2 to 3 experiments with 10–15 cells analyzed per experiments. ***p < 0.001; One way ANOVA with Bonferroni’s post-test. Method details for these assays (plasmid construction, transfection, and immunofluorescence confocal microscopy) can be found in Avia et al. (2019). For NS3 pixel colocalization analysis with HGS or LAMP-1 signal, z-stack images (step 0.5 μm) of transfected cells were captured for each channel, cell mask obtained (Mulens-Arias et al., 2015), positive signal for each channel was determined with the ImageJ threshold tool and percentage of overlapping pixels in cell masks for each z-plane determined with the ImageJ image calculator tool (“AND” operation). Manders’ coefficient in cell masks was obtained using the JACOP ImageJ plugin (Bolte and Cordelieres, 2006).

NS3 has also been shown to induce autophagic degradation of STAT2 thereby inhibiting IFN-I signaling (Avia et al., 2019). In infection with BTV lacking the segment encoding for NS3, STAT2 degradation did not occur demonstrating that NS3 is central to this mechanism. By mutating NS3 lysine residues 13 and 15, Avia et al. (2019) also showed that NS3 is ubiquitinated and that ubiquitination on both residues is essential for NS3 targeting of STAT2 to autophagic degradation. Moreover, the NS3 mutant unable to be ubiquitinated was not capable of inhibiting IFN signaling in reporter assays, which indicated that NS3 ubiquitination is critical for its interference in IFN signaling. NS3 appeared to be polyubiquitinated with K63-linked chains. In mammals, post-translational modification of proteins with ubiquitin linked through the K63 isopeptidic bonds is associated, among other things, with endocytosis and vesicular sorting in the multivesicular bodies (MVB) (Lauwers et al., 2009; Erpapazoglou et al., 2014). It could therefore be speculated that NS3 ubiquitination allows NS3 sorting within the cellular vesicular system. In immunofluorescence studies, we found that the NS3 mutant lacking ubiquitination sites did not colocalize with the MVB marker HGS (Figure 5), confirming the importance of this post-translational modification for NS3 traffic and function. NS3 also possesses an E3 ligase recruitment site in its N-terminal region that is involved in virus release (Wirblich et al., 2006). Avia et al. (2019) also showed that this domain was important for NS3-mediated autophagic degradation of STAT2. This same mutant also failed to colocalize with the MVB marker HGS (Figure 5), indicating that this domain must remain intact for NS3 correct vesicular traffic.

NS3 is therefore a polyfunctional protein encoded by BTV. It allows virion egress in insect and mammalian cells, but it also interferes with IFN induction and signaling. It appears that NS3 ubiquitination through K63 chains could be central to its trafficking through the vesicular cellular system, thus giving NS3 access to cellular location necessary to IFN induction like the Golgi apparatus where it impairs TBK1 activation, and to degradative pathways, such as the autophagic/lysosomal pathway through which it mediates STAT2 degradation. Further understating of NS3 biology will help shed more light on the exact mechanisms of interference that this protein mediates.

### BTV-NS4

BTV NS4 protein was first proposed as an overlapping ORF by bioinformatics analysis (Firth, 2008) and subsequently described in 2011 (Belhouchet et al., 2011; Ratinier et al., 2011). NS4 is encoded by an alternative open reading frame in segment 9. NS4 expression can be detected in the cytoplasm as well as in the nucleus of BTV-infected cells. NS4 putative coiled-coil structures indicate that it can be associated with nucleic acids and/or membranes, and indeed it has been shown to localize with lipid droplets in the cytoplasm and with the nucleoli in the nucleus (Belhouchet et al., 2011). NS4 contains a sequence of basic amino-acid residues that drives nuclear localization and two putative leucine zipper domains (Ratinier et al., 2011). NS4 has been shown to bind to dsDNA (Belhouchet et al., 2011). Ratinier et al. (2011) showed that NS4 expression is not essential for BTV replication and confirmed the localization of NS4 in the nucleoli during infection. A reverse genetic BTV-8 lacking NS4 was attenuated in sheep when compared to infections with a counterpart that expressed the protein (Ratinier et al., 2016), thus indicating that NS4 modulates host immune responses.

Indeed, early studies already indicated that NS4 could interfere with the IFN response, thus providing a replicative advantage to BTV (Ratinier et al., 2011). NS4 can antagonize IFN induction and IFN-I and-II signaling (Ratinier et al., 2016; Avia et al., 2019; Li et al., 2021). Transcriptomic analysis showed that 102 genes related to the IFN pathways were upregulated in infections with the NS4 defective virus when compared to the NS4-expressing counterpart (Ratinier et al., 2016). In reporter assays, NS4 was capable of inhibiting not only the IFN-β promoter and ISRE-containing promoters but also the CMV promoter (albeit to a lower extent) indicating that NS4 could alter the activity of a wide range of promoters (Ratinier et al., 2016). NS4 cloned from different BTV strains (including the atypical BTV-25 and -26) could impair gene expression under the control of a CMV promoter, except for one clone that lacked the basic amino-acid sequence responsible for nuclear localization (Ratinier et al., 2016). This further confirms that one of NS4 function during BTV infection is likely to involve modulation of host gene expression including IFNs and ISGs after translocation in the nucleus.

NS4 has also been shown to impair IFN-I and-II signaling in a dose dependent manner in reporter assays (Avia et al., 2019). Expression of NS4 alone does not affect STAT1 phosphorylation after IFN stimulation (Avia et al., 2019; Li et al., 2021); however, its co-expression with NS3 appears to enhance NS3 capacity to impair STAT1 phosphorylation (Li et al., 2021). While Avia et al. (2019) did not detect NS4 interaction with STAT1; Li et al. (2021) reported that NS4 interacted with the SH2 domain of STAT1. As for NS3 immunoprecipitation assays in these two studies, these discrepancies could be due to differences in experimental procedures, since in Avia et al. (2019) work interaction was assessed using endogenous STAT1 levels, while Li et al. (2021) used STAT1 transfection. Alternatively difference in NS4 activity between the different BTV strains used in these studies could also contribute to these observations. Indeed, Ratinier et al. (2011) showed that NS4 activity on the IFN response varied between BTV serotypes. The expression of NS4 protein from BTV-8 allowed BTV replication in IFN-treated cells, whereas NS4 from BTV-1 did not potentiate this effect (Ratinier et al., 2011), which indicated that NS4 activity on the IFN response can vary depending on the BTV isolates. Li et al. (2021) also showed that formation of the complex NS3 + NS4 + STAT1 reduced the heterodimerization of STAT1 and STAT2 that is necessary for IFN-I signaling. Co-transfection of NS3 and NS4 also impaired the translocation to the nucleus of phosphorylated STAT1 further confirming that NS4 could potentiate NS3 effects on STAT1 activity (Li et al., 2021). The mechanism through which the sole expression of NS4 mediates IFN signaling inhibition has, however, yet to be elucidated. Whether this involves interaction with components or adaptors critical to IFN signaling and/or interference with ISG transcription is an open question.

All studies so far concur in showing that NS4 is an antagonist of IFN induction and signaling. NS4 is likely to modulate gene expression in the nucleoli and could affect IFN signaling when present in the cytoplasm. Its conservation throughout BTV serotypes and in other orbiviruses (Belhouchet et al., 2011; Ratinier et al., 2011, 2016; Zwart et al., 2015; Boughan et al., 2020), and the observation that its deletion leads to attenuation also indicates that it provides the virus with a replicative advantage.

### The Role of Other BTV Proteins

There are indications that NS3 and NS4 are not the only BTV proteins involved in IFN antagonism. Using reporter assays, VP3 and VP4 were shown to interfere with IFN induction (Chauveau et al., 2013). NS1 and NS2 were also shown to inhibit IFN induction in reporter assays (Stewart and Roy, 2010), although this was not confirmed in other studies (Chauveau et al., 2013; Avia et al., 2019). Several factors could account for these discrepancies, such as the difference in the reporter system used as well as the diverse stimuli used for IFN induction (poly I:C transfection (Stewart and Roy, 2010), constitutively active RIG-I transfection (Chauveau et al., 2013), or Sendai virus infection (Avia et al., 2019).

Recently, the interference with IFN induction mediated by the inner core protein VP3 was further characterized (Pourcelot et al., 2021). VP3 is important in virus assembly and has been shown to interact with NS2 in virus inclusion bodies (Kar et al., 2005; Mohl and Roy, 2014). VP3 could impair the IFN-β promoter activity in a dose dependent manner in reporter assays, and limit ISG56 induction (Pourcelot et al., 2021). VP3 was shown to interact with MAVS and IKKε downstream of RIG-I activation (Pourcelot et al., 2021). More precisely, Pourcelot et al. (2021) mapped the interaction of VP3 to the C-terminal region of MAVS. This region contains a TRAF-interacting motif required for the correct activation of the IFN response as well as a regulatory binding site that recruits IKKε after MAVS ubiquitination on lysine 500 (Paz et al., 2011). The exact mechanism of VP3 action on this pathway is not fully elucidated. It could be speculated that VP3 impairs TRAF3 recruitment to MAVS and/or recruit IKKε regulatory activity to dampen IFN induction. Further studies will be necessary to understand this interaction of BTV with the IFN induction pathway.

As previously stated VP4 was shown to interfere with IFN induction in reporter assays (Chauveau et al., 2013). This was not observed in a separate study (Stewart and Roy, 2010) though it should be noted that a different strategy was used for IFN stimulation. The VP4 capping enzyme possesses a nucleoside-2′-O-methyltransferase enzyme activity that is essential to virus replication (Stewart and Roy, 2015). 2′-O-methylase enzymatic activity has also been shown to allow evasion of viral RNA recognition by PRRs, such as IFIT family members or TLR7 (Daffis et al., 2010; Jöckel et al., 2012). In rotavirus, another member of the *Reoviridae* family, the VP3 capping enzyme possesses a 2′-O-methyltransferase activity that limits viral RNA recognition by cytosolic sensors (Chen et al., 1999; Uzri and Greenberg, 2013). Whether BTV VP4 capping enzyme acts similarly will require further work.

Finally a putative ORF has been described in segment 10 that could encode for a fifth non-structural protein (NS5) (Stewart et al., 2015). Although evidence of its expression during BTV infection has yet to be established, the conservation of this alternative ORF within segment 10 in more than 350 BTV sequences, as well as its presence in other orbiviruses (such as AHSV) indicates that this ORF probably encodes for a protein (Stewart et al., 2015). Stewart et al. (2015) also showed using a segment 10 construct that a luciferase reporter gene could be translated by the alternative ORF providing further evidence that this alternative ORF could encode for a non-structural protein. The putative NS5 protein possesses a nucleolar localization signal, and immunofluorescence studies showed its localization in this nuclear space (Stewart et al., 2015). In reporter assays, NS5 inhibited gene expression driven by several promoters including the IFN-β promoter, and this was dependent on the nucleolar localization signal. However reverse genetics BTV-8 lacking the putative ORF induced similar IFN levels as its counterpart with the ORF, suggesting that the putative NS5 is not an IFN antagonist. This reverse genetic mutant also had similar virulence as the wild-type virus in murine models (Stewart et al., 2015). The nucleolar localization of NS5, its capacity to impair gene expression, and its conservation in BTV sequences does nonetheless suggest that it could have relevance in the cellular shutoff produced by BTV (Huismans, 1971). NS4 also localize to the nucleoli and affect gene expression driven by several promoters (Ratinier et al., 2016). It is tempting to speculate that both proteins could participate in this phenomenon that is likely to contribute to BTV evasion from antiviral mechanisms.

### Vector Influence on the IFN Response

An often overlooked aspect of BTV infection is the effects mediated by the vector on immunity, as these are difficult to quantify. Interestingly, the IFN response differed in sheep experimentally infected with BTV-carrying *Culicoides* spp. or needle-injected with the virus (Pages et al., 2014). Infection with *Culicoides* spp. delayed the IFN response and the production of neutralizing antibodies when compared to infection through needle injection. Indeed, the amount of local inflammation due to the *Culicoides* spp. bites appear to inversely correlate with viremia load in infected sheep (Pages et al., 2014). It has also been described that infections with tissue cultured BTV can differ from direct infection with blood-infected animals in terms of virulence (MacLachlan et al., 2008; Eschbaumer et al., 2010; Caporale et al., 2014), and this has been correlated to decreased variability in the BTV variant population passaged in tissue culture in mammalian cells (Caporale et al., 2014). RNA viruses never exist as a single genotype but rather as a range of variants (or quasispecies although to our knowledge this concept has not been directly studied in BTV). Increased variability could facilitate the adaptation of BTV to external pressure. Interestingly while the consensus sequence of BTV passaged in mammalian cells or in a *Culicoides* spp. cell line was maintained, passage in the *Culicoides* spp. cell line augmented the amount of variants (Caporale et al., 2014). The interplay between host and vector factors can clearly influence BTV pathogenesis and by extension the early recognition steps of the virus by the immune system. Multiple factors probably participate in this effect, such as *Culicoides* spp. saliva products that limit inflammation or the diversity of variants generated in the insect host. It would be interesting for BTV research to establish experimental infection models in ruminants that better mimic the field conditions so that a more in depth understanding of BTV interaction with the early events of immune recognition can be studied.

## Conclusion

Although BTV was traditionally seen as a good inducer of IFN, it is now evident that it possesses multiple mechanisms to impair the IFN response and consequently promotes its replication. The non-structural proteins NS3 and NS4 have emerged as the main IFN antagonists in BTV infections, but recent studies have also shown that structural proteins like VP3 can modulate the IFN response. The interaction of BTV proteins with the IFN system is also central to the virus pathogenicity, and could therefore help advance vaccine design. Understanding these cellular virus-host interactions will also shed some light on the polyfunctionality that some BTV gene products display. Ultimately, studying BTV interaction with the IFN system will help to further comprehend the complex biology that takes place during BTV infections.

## Author Contributions

All authors made a direct intellectual contribution to the work. JR wrote the main body of the manuscript. MA and JR designed the figures. All authors contributed to manuscript revision, read, and approved the submitted version.

## Conflict of Interest

The authors declare that the research was conducted in the absence of any commercial or financial relationships that could be construed as a potential conflict of interest.
